# Supplementary material for: Bioinspired Cyclic Dipeptide Functionalized Nanofibers for Thermal Sensing and Energy Harvesting
Source: Materials (Basel). 2023 Mar 21;16(6):2477. doi: 10.3390/ma16062477 (PMC10055687; doi:10.3390/ma16062477)
Supplement: Supplementary file 1 [file materials-16-02477-s001.zip › materials-2263995-supplementary.pdf]

materials

Bioinspired cyclic dipeptide functionalized nanofibers for thermal sensing and energy harvesting

Daniela Santos [1], Rosa M. F. Baptista [1,*], Adelino Handa [1], Bernardo Almeida [1], Pedro V. Rodrigues [2], Ana R. Torres [2], Ana Machado [2], Michael Belsley [1] and Etelvina de Matos Gomes[1]

[1] Centre of Physics of Minho and Porto Universities (CF-UM-UP), University of Minho, Campus de Gualtar, 4710-057 Braga, Portugal

[2] Institute for Polymers and Composites, University of Minho, Campus de Azurém, 4800-058 Guimarães

*Corresponding author: rosa_batista@fisica.uminho.pt

Supplementary Information

S1. Optical Microscopy

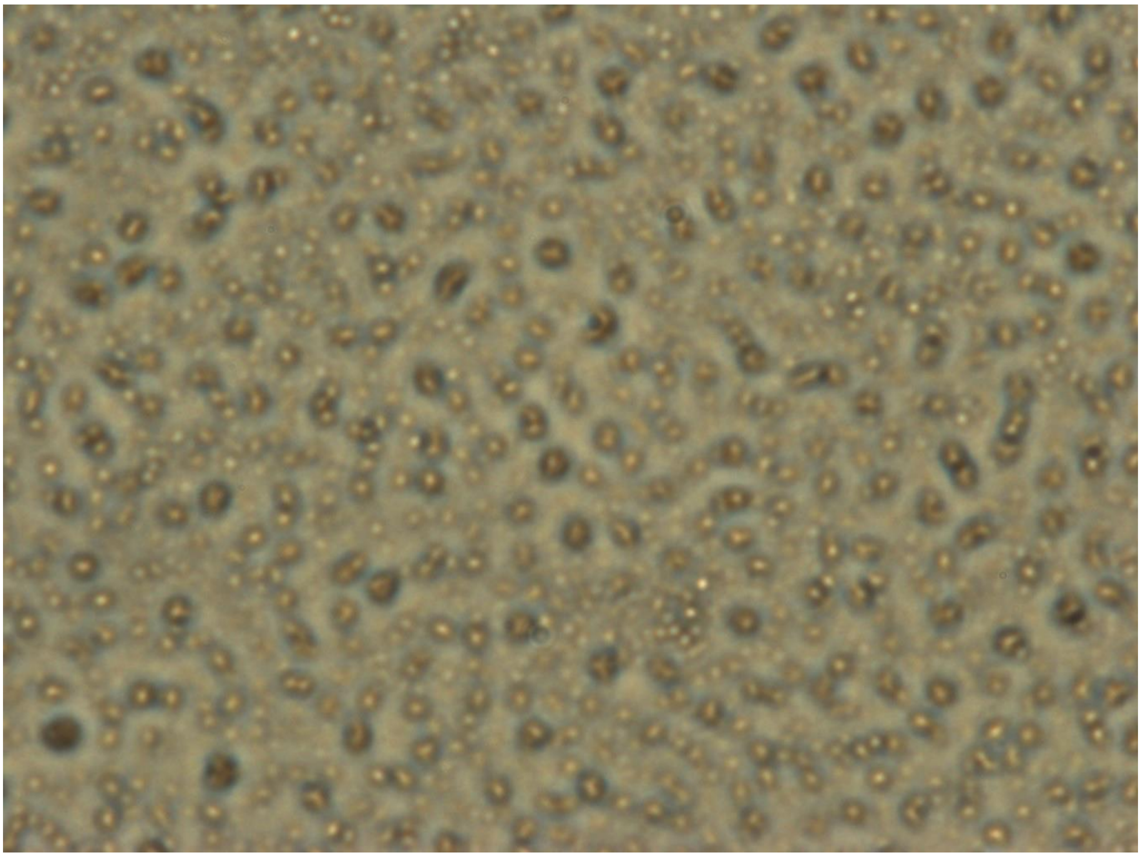

Figure S1. Optical microscopy image at 1000x magnification of Cyclo (L-Trp-L-Trp) nanospheres formed in a methanol solution with a concentration of 2 mg/mL.

S2. Calculation of quantum dots (QD) radius

The calculated radius of a QD for Cyclo (L-Trp-L-Trp) is $1.41\ nm$ based on a model of an organic QDs, given by the equation,

$$R = \pi r_B^0 \sqrt{\frac{m_0/M}{\frac{\mu}{m_0 \varepsilon_\infty^2} - \frac{E_{ex}^{QD}}{R_y}}}$$

according to [1]. In the above equation, $R_y = 13{,}56\ eV$ is the Rydberg constant, the Bohr atomic radio of the hydrogen atom is r_B^0=0.53 Å. The free electron mass is $m_0 = 9.11 \times 10^{-31}$ kg, the exciton mass is $M \sim m_0$ the reduced mass of the electron-hole pair (exciton) is μ ~0.25m_0 and $\varepsilon_\infty^2 = 2.25$ assumed for peptide QDs.
From the measured optical absorption spectra, absorption starts at $\lambda_i = 247\ nm\ (5.02\ eV)$ indicating the breaking of the exciton binding state. The binding energy of the exciton is $E_{ex}^{QD} = 0.48\ eV$, the difference between the phononless line at $\lambda_g^0 = 273\ nm\ (4.54\ eV)$ and λ_i.

S3. Tauc Plot

Tauc plot is a method used to determine the optical band gap of nanoparticles from UV-vis absorption spectroscopy.

The equation,

$$(\alpha h\nu)^n = K(h\nu - E_g) \qquad (1)$$

is known as Tauc relation. In this equation, α is the absorption coefficient, $h\nu$ is the incident photon energy, K a constant of proportionality independent of the energy (for amorphous materials, K= 1), and E_g is the band gap energy of the nanomaterial. The exponent n represents the type of electronic transition and for this work, $n = {}^1/_2$ as this is an indirect band gap.

In this method, energy is plotted on x-axis and $(\alpha h\nu)^n$ on the y-axis. Then draw the tangent line on the curve where $\alpha = 0$. The point where it connects with the x-axis is the optical band gap energy, E_g (as seen in inset, Figure 10).

In UV-vis absorption spectroscopy, the experimental data includes the wavelength, λ, and absorbance, A.

From the wavelength, it is possible to calculate the energy:

$$E = h\nu = \frac{hc}{\lambda} \approx \frac{1240\ (eV\ nm)}{\lambda\ (nm)}$$

To determine the absorption coefficient, α, from the absorbance, A:

$$A = \log_{10}\left(\frac{I_0}{I}\right) = \alpha l \log_{10} e \Leftrightarrow \alpha \approx 2{,}303\ A\ (cm^{-1})$$

Here, I_0 is the incident intensity and I the transmitted intensity, with path length $l = 1\ cm$.

From equation (1) and making the necessary alterations:

$$(\alpha h\nu)^2 = (2{,}303\ A\ h\nu)^2 (eVcm^{-1})^2$$

S4. Kubelka-Munk Function

The Kubelka-Munk function gives the ratio of absorption and scattering constants, where R is the percentage of reflectance given by the experimental data:

$$F(R) = \frac{(1-R)^2}{2R}$$

To calculate the band gap energy from UV-vis reflection (Diffuse Reflectance Spectroscopy) data, for materials with indirect band gap:

$$\left(h\nu\, F(R)\right)^{1/2} = \alpha\left(h\nu - E_g\right)$$

S5. Piezoelectric nanogenerator

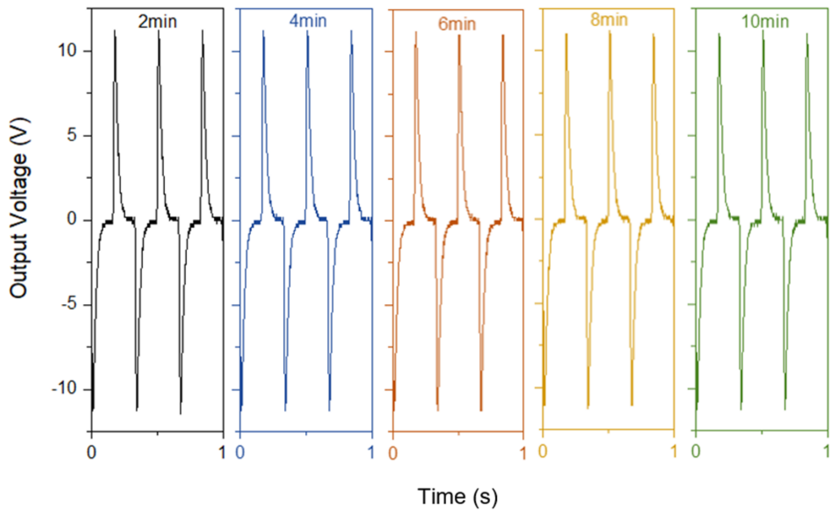

Figure S2. Output voltage as a function of time from Cyclo (L-Trp-L-Trp) incorporated into PLLA electrospun nanofibers.

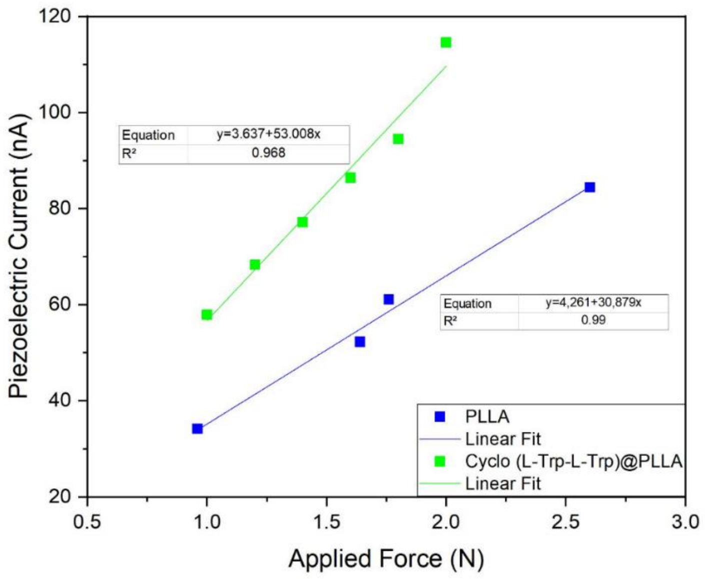

Figure S3. Piezoelectric current versus applied force for Cyclo (L-Trp-L-Trp)@PLLA and PLLA neat fibers, with the respective linear fits.

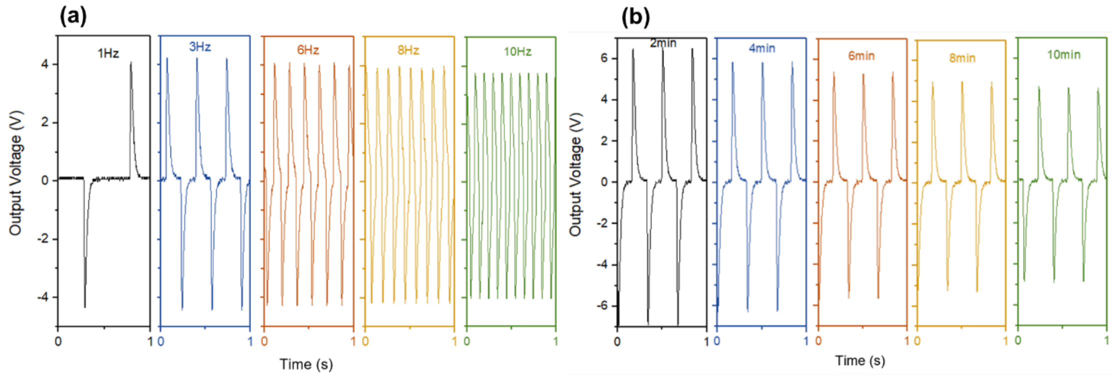

Figure S4. (a) Output voltage for low frequencies up to 10 Hz; **(b)** Output voltage as a function of time, from Cyclo (L-Trp-L-Trp) incorporated into PCL electrospun nanofibers.

Reference

1. Amdursky, N.; Molotskii, M.; Gazit, E.; Rosenman, G. Self-assembled bioinspired quantum dots: Optical properties. *Appl. Phys. Lett.* **2009,** *94*, 261907.
